# *PLOS Pathogens *expanding horizons

**DOI:** 10.1371/journal.ppat.1012278

**Published:** 2024-06-13

**Authors:** Julia Squarr, Rebecca Kirk, Alexander E. Gorbalenya, Michael Malim

**Affiliations:** 1 PLOS, Berlin, Germany; 2 PLOS, Cambridge, United Kingdom; 3 Department of Medical Microbiology, Leiden University Medical Center, Leiden, the Netherlands; 4 Department of Infectious Diseases, School of Immunology and Microbial Sciences, King’s College London, London, United Kingdom

*PLOS Pathogens* is, first and foremost, a community-driven journal focusing on microbes. The field and our community are growing and ever-changing, and *PLOS Pathogens* is therefore committed to adapting and evolving to meet the demands and wishes of our community who work on, and follow, pathogen-related research.

*PLOS Pathogens* acknowledges these shifts, and in this Editorial, we draw attention to the journal’s revised scope statement and highlight our commitment to reporting outstanding fundamental discovery, translational, or applied research—encompassing all pathogens and life forms. This strategic evolution reflects our recognition of the pressing societal need to translate scientific insights into tangible solutions that address real-world challenges and opportunities. By empowering scientists and the public with an open science communication platform, *PLOS Pathogens* seeks to ensure that research on microbes goes beyond the laboratory.

The SARS-CoV-2/COVID-19 pandemic serves as a beacon for the real-world impact of timely translational research. The rapid development of diagnostics, therapeutics, and vaccines during this global crisis exemplifies the benefits of connecting fundamental and translational research for deliverable outcomes. Researchers, stakeholders, and knowledge sharing institutions like *PLOS Pathogens* continue to draw lessons from this unprecedented experience.

Here, the *PLOS Pathogens’* Editors reaffirm our responsibility to support research excellence along the complete research pathway. We continue to welcome studies that are not purely observational but provide significant advances to our understanding of microbe biology and pathogenesis and may also extend far beyond the laboratory, impacting the clinic, the field, and everyday life.

In line with this commitment, we’ve recently broadened our article formats for submission, now welcoming Short Reports alongside our established Research Articles. The objective of the Short Reports is to encourage the concise communication of exciting and impactful discoveries.

As we embark on this transformative journey, we invite our readers, authors, researchers, and clinicians across disciplines to join us in shaping the future.

